# Alternative and facile production pathway towards obtaining high surface area PtCo/C intermetallic catalysts for improved PEM fuel cell performance†

**DOI:** 10.1039/d2ra07780a

**Published:** 2023-02-06

**Authors:** Philipp A. Heizmann, Hien Nguyen, Miriam von Holst, Andreas Fischbach, Mitja Kostelec, Francisco Javier Gonzalez Lopez, Marjan Bele, Luka Pavko, Tina Đukić, Martin Šala, Francisco Ruiz-Zepeda, Carolin Klose, Matija Gatalo, Nejc Hodnik, Severin Vierrath, Matthias Breitwieser

**Affiliations:** a Electrochemical Energy Systems, IMTEK – Department of Microsystems Engineering, University of Freiburg Georges-Koehler-Allee 103 79110 Freiburg Germany matthias.breitwieser@hahn-schickard.de; b Institute and FIT – Freiburg Center for Interactive Materials and Bioinspired Technologies, University of Freiburg Georges-Köhler-Allee 105 79110 Freiburg Germany; c Hahn-Schickard Georges-Koehler-Allee 103 79110 Freiburg Germany; d Department of Materials Chemistry, National Institute of Chemistry Hajdrihova ulica 19 1000 Ljubljana Slovenia; e ReCatalyst d.o.o. Hajdrihova ulica 19 Ljubljana 1000 Slovenia; f Department of Analytical Chemistry, National Institute of Chemistry Hajdrihova ulica 19 1000 Ljubljana Slovenia

## Abstract

The design of catalysts with stable and finely dispersed platinum or platinum alloy nanoparticles on the carbon support is key in controlling the performance of proton exchange membrane (PEM) fuel cells. In the present work, an intermetallic PtCo/C catalyst is synthesized *via* double-passivation galvanic displacement. TEM and XRD confirm a significantly narrowed particle size distribution for the catalyst particles compared to commercial benchmark catalysts (Umicore PtCo/C). Only about 10% of the mass fraction of PtCo particles show a diameter larger than 8 nm, whereas this is up to or even more than 35% for the reference systems. This directly results in a considerable increase in electrochemically active surface area (96 m^2^ g^−1^*vs.* >70 m^2^ g^−1^), which confirms the more efficient usage of precious catalyst metal in the novel catalyst. Single-cell tests validate this finding by improved PEM fuel cell performance. Reducing the cathode catalyst loading from 0.4 mg cm^−2^ to 0.25 mg cm^−2^ resulted in a power density drop at an application-relevant 0.7 V of only 4% for the novel catalyst, compared to the 10% and 20% for the commercial benchmarks reference catalysts.

## Abstract 2

A novel PtCo/C based PEMFC electrocatalyst was investigated in real fuel cells under application-relevant conditions. The corresponding MEAs show superior performance compared to reference materials due to more suitable nanoparticle sizes.

## Graphical abstract

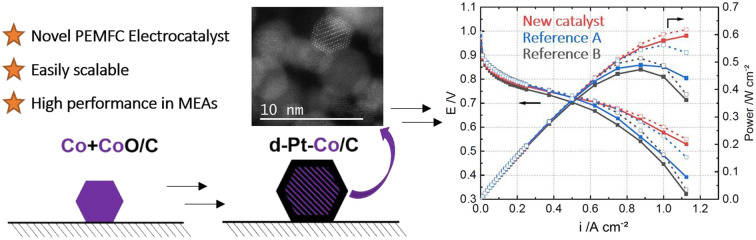

## 1 Introduction

On the way to developing and implementing sustainable clean energy technologies to combat climate change and pollution, proton exchange membrane fuel cells (PEMFCs) present themselves as promising technology for power source applications in the automobile and energy industries due to their high power density, low operating temperature and fast refuelling times.^1^ A major barrier to large-scale commercialization is the high cost of PEMFCs, with the common platinum-based catalyst accounting for a substantial portion of the price. At high production volume (500 k systems/annually), the platinum-based catalyst represents >40% of the total system costs, which will not benefit from the economies of scale.^2–4^ Thus, while economies of scale in general will be a crucial cost reduction driver, it is also critical to reduce the required amount of noble metals in the PEMFC.

Numerous studies showed that PtM/C alloy catalysts (M *e.g.* Co, Ni, Cu, Fe) exhibit an increased activity for the oxygen reduction reaction (ORR) and thus lead to a remarkable performance improvement of the corresponding membrane electrode assembly (MEA) in PEMFCs compared to pure Pt/C based systems.^5–10^ Advantageously, the dilution of the particle core with these 3d transition metals simultaneously leads to a reduction in the total amount of noble metal.^11^ However, the use of these alloying transition metals also comes with two major disadvantages: the higher complexity of the overall catalyst, *e.g.* regarding the more difficult synthetic access, and the intrinsic thermodynamic instability of the alloy catalysts under acidic conditions.^12^ One approach to the latter problem is to selectively deplete the transition metal concentration at the particle surface to practically prevent the dissolution while preserving the positive influences of alloy formation (ligand and crystal strain effects) as much as possible. For example, PtCo nanoparticles with Pt-rich shell of ∼3 atomic layers still exhibits significantly improved ORR activity.^13,14^ The process of Pt-rich shell formation (in other words, depletion of M from the PtM shell) can be accomplished by electrodissolution or by washing in acidified solutions.^15–17^

In addition to fundamental research focusing on improving the performance and stability of the catalysts, studies on optimization and scalable synthesis routes are as important.^17–22^ In the last years, some research has tackled this topic. For instance, as part of our recent work we reported a novel double passivation galvanic displacement method for Pt-alloy catalysts with high reproducibility and great flexibility allowing a highly targeted catalyst design, where the chemical composition and loading of the alloy on the carbon support can be tuned very precisely.^23^ This approach can potentially be applied to a wide range of sacrificial metals M and on a variety of carbon supports while allowing the production of the resulting catalyst on a multigram scale.^24^ In these studies, the reported synthesized catalysts showed promising electrochemical improvements in both rotating-disk electrode (RDE) and gas-diffusion electrode (GDE) tests compared to commercially available catalysts, including higher specific activities (SA), mass activities (MA) and electrochemical surface areas (ECSA).

In the present study, we take advantage of the promising catalytic activity for the ORR and the high ECSA of the Pt-alloy catalyst based on double passivation galvanic displacement.^23–25^ We confirm the significantly narrower particle size distribution of the novel PtCo/C catalysts compared to commercial PtCo/C catalysts *via* transmission scanning electron microscopy (TEM) image analysis and X-ray powder diffraction (XRD). In addition, the effect of the significantly narrowed particle size distribution obtained by the optimized synthesis route on the performance is demonstrated with thin-film rotating disk electrodes (TF-RDEs) and in single-cell MEA validation.

## 2 Experimental

### 2.1 Catalyst synthesis

**Scheme 1:**
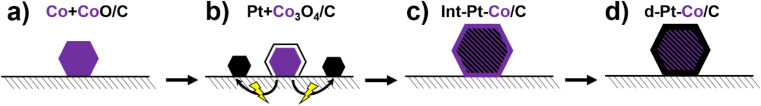
Alternative preparation method of de-alloyed intermetallic PtCo/C catalysts *via* the 4 step process of using (a) sacrificial Co-based precursor, (b) double passivation galvanic displacement to deposit Pt NPs, (c) thermal annealing to obtain the intermetallic crystal structure as well as (d) de-alloying to obtain the final catalyst material.

The synthesis of the new experimental PtCo/C catalyst (hereafter referred to as ReCatalyst) was based on the previous works and can be conceptualized into four main steps (Scheme 1).

#### 2.1.1 Double passivation with galvanic displacement of Co+CoO/C precursor with a Pt-salt

In the second step (Scheme 1a and b), Pt NPs were deposited onto the carbon support (Ketjenblack EC300J) *via* previously reported double passivation galvanic displacement method.^23–25^ Briefly, the Pt deposition step consists of oxide passivation of Co followed by carbon monoxide (CO) capping of Pt-based NPs formed by galvanic displacement of Co after Pt-salt addition (Scheme 1b). To achieve that, multigrams of proprietary Co+CoO/C composite (Scheme 1a), prepared using the pulse combustion method reported elsewhere,^24^ were suspended in an aqueous solution. The suspensions were then ultrasonicated (Ultrasound bath Iskra Sonis 4) for 3 minutes (degassing). Afterwards, the suspensions were first purged with Ar and then switched to CO. 0.1 M K_2_PtCl_4_ (Apollo scientific) was added to the CO-saturated suspension of Co/C composite while continuously purging the reaction mixture with CO. After the entire Pt-salt solution was added to the reaction mixture, the suspension was filtered and washed with Milli-Q water 3 more times. The obtained composites were left to dry at 50 °C overnight.

#### 2.1.2 Formation of the intermetallic Pt-alloy

In the second step, Pt and Co metals were alloyed *via* high-temperature thermal annealing of the obtained Pt+Co_3_O_4_/C composite (Scheme 1b and c). All composite powders were placed in a Al_2_O_3_ crucible in a separate experiment due to different thermal annealing conditions. The crucibles were then put into a quartz tube that was sealed and purged with Ar for 2 hours in order to ensure an inert atmosphere prior to raising the temperature to 700 °C with a ramp of 10 K min^−1^ for 24 h. Afterwards, the furnace was cooled with a ramp of 10 K min^−1^ to 600 °C for another 24 h for the formation of the PtCo intermetallic phase, followed by cooling to RT with a ramp of 10 K min^−1^. In the case of all experiments, the quartz tubes were purged with Ar for the entire duration of the thermal annealing process.

#### 2.1.3 De-alloying

The PtCo catalyst was subjected to the same activation (acid washing; Scheme 1c and d) protocol reported elsewhere.^26,27^ Briefly, the process involves a 24 h washing in 0.5 M H_2_SO_4_ at 80 °C. Afterward, the catalysts were washed 4 times with Milli-Q water (18.2 MΩ cm^−1^).

### 2.2 Characterization

#### 2.2.1 Inductively coupled plasma optical emission spectrometry (ICP-OES)

All reagents used were of analytical grade or better. For sample dilution and preparation of standards, ultrapure water (18.2 MΩ cm^−1^, Milli-Q, Millipore) and ultrapure acids (HNO_3_ and HCl, Merck-Suprapur) were used. Standards were prepared in-house by dilution of certified, traceable, inductively coupled plasma (ICP)-grade single-element standards (Merck CertiPUR). A Varian 715-ES ICP optical emission spectrometer was used. Before ICP-OES analysis, each catalyst powder was weighted (approximately 10 mg) and digested using a microwave-assisted digestion system (Milestone, Ethos 1) in a solution of 6 mL HCl (conc.) and 2 mL HNO_3_ (conc.). Samples were then filtered, and the filter paper was again submitted to the same digestion protocol. These two times digested samples were cooled to room temperature and then diluted with 2% v/v HNO_3_ until the concentration was within the desired concentration range.

#### 2.2.2 X-ray powder diffraction (XRD)

All X-ray diffractograms were acquired on a X'Pert PRO diffractometer (Malvern Panalytical) with Cu Kα radiation (*λ* = 1.541874 Å). The powder samples were prepared on a zero-background Si holder and measured in the 2*θ* range from 10° to 60° with a step size of 0.039° per 300 s by using a fully opened Pixcel detector.

#### 2.2.3 Scanning transmission electron microscopy (S/TEM)

TEM micrographs were recorded on a Talos F200X (S)TEM (ThermoFisher, high-brightness X-FEG emitter) at 200 kV acceleration voltage with a Ceta 16 Megapixel CMOS Camera. Samples were dispersed in isopropyl alcohol, briefly sonicated (Bandelin Sonorex super RK 100 H) and loaded onto copper TEM grids (carbon film, 3–4 nm nominal thickness, 200 hexagonal mesh or lacey/carbon film, 200 quadratic mesh, both ScienceServices GmbH). The TEM grids were dried in air and mounted on a model 2020 tomography holder (Fischione Instruments). Due to synthetically based chemical and structural inhomogeneities in the carbon support the nucleation and growth behaviour of the nanoparticles may differ slightly depending on the carbon primary particle. Therefore, a minimum of 2000 nanoparticles on at least 10 carbon particles per sample were considered to enable quantitative conclusions. Nanoparticle size distributions for each sample were determined *via* ImageJ 1.53c. Tilt series were acquired automatically over a 140–144° tilt range (±70–72°, 1° tilt increment) in STEM imaging mode with a beam current of ∼50 pA and a convergence angle of ∼9 mrad using the tomography STEM software V4.20. The collected Bright-field (BF) and high-angle annular dark-field (HAADF) image pairs (1024 × 1024 pixels, 15 μs dwell time) were binned by a factor of 2 and aligned by cross-correlation using Inspect3D. Energy dispersive X-ray (EDX) mappings were acquired in STEM-mode with a field of view of 100 nm. The acquisition was performed with 100 scans and 10 μs dwell time per scan (total acquisition time ∼ 20 min).

Aberration-corrected STEM (Cs-STEM) micrographs were acquired using a JEM-ARM200CF (JEOL Ltd, Cold FEG emitter) at 80 kV acceleration voltage with a convergence angle of 25 mrad.

#### 2.2.4 Scanning electron microscopy (SEM)

The catalyst layer thickness was determined *via* SEM micrographs of cryo-cut cross-sections of the MEAs. The samples were cut in liquid nitrogen and mounted on standard aluminium 90° SEM Stubs (ScienceServices GmbH) with conductive double-sided adhesive carbon tabs. The SEM micrographs were acquired using a FE-SEM Amber X (Tescan GmbH) equipped with a secondary electron detector (Everhart-Thornley type). All micrographs were acquired at a working distance of approximately 6 mm using an acceleration voltage of 2 kV and a beam current of 100 pA. SEM micrographs and additional information regarding the measurement are presented in the ESI.†

#### 2.2.5 Thin-film rotating disk electrode

Oxygen reduction reaction (ORR) polarization curves and carbon monoxide (CO) electrooxidation cyclic voltammetry (CV) were measured in a thin-film rotating disk electrode (TF-RDE), of which the setup was described in the previous works.^25,28^

As a reference electrode (RE), the reversible hydrogen electrode (RHE; HydroFlex, Gaskatel) was used, while the graphite rode electrode was used as a counter electrode. The working electrode (WE) was a 0.196 cm^2^ glassy carbon disc embedded in Teflon (Pine Instruments). The WE was prepared following the procedure, which was also reported in the previous work:^28^

- Polishing to a mirror finish with Al_2_O_3_ paste (particle size 0.05 μm, Buehler) on a polishing cloth (Buehler).

- Rinsing and ultrasonication (Ultrasound bath Iskra Sonis 4) in Milli-Q water for 5 min.

- Pipetting 20 μl of a freshly prepared water-based catalyst ink (1 mg_catalyst_ per 1 ml_Milli-Q water_) on the WE so that the WE is completely covered by the dispersion.

- After the drop had dried, 5 μl of Nafion solution (EelctroChem, 5% aqueous solution) diluted in isopropanol (1 : 50 v/v) was added to the WE with the catalyst ink to bind the catalyst thin-film on the glassy carbon electrode.

After preparing the WE, it was mounted on the rotator (Pine Instruments). The RDE measurements performed in 0.1 M HClO_4_ are as follows: ORR polarization curves were measured in an oxygen saturated electrolyte with rotation at 1600 rpm between 0.05 and 1.0 V_RHE_ with a scan rate of 20 mV s^−1^. After the ORR polarization curve measurement, the electrolyte (0.1 M HClO_4_) was purged with carbon monoxide (CO) under potentiostatic mode (0.05 V_RHE_) for CO-stripping experiments, followed by Ar to saturate the electrolyte. CO-electrooxidation (“stripping”) was performed using the same potential window (0.05–1.0 V_RHE_) and scan rate (20 mV s^−1^) as in ORR polarization curves, but without rotation and in an Ar-saturated electrode.^29^

Kinetics parameters from RDE experiments were calculated at 0.9 V_RHE_ with Koutecky–Levich equation, as described in literature^30,31^*i*^−1^ = *i*_k_^−1^ + *i*_d_^−1^where *i* is the measured current density, *i*_k_ is the kinetic current and *i*_d_ is the diffusion-limited current. From the kinetic current, the mass activity (MA) can be obtained by normalizing the kinetic current *i*_k_ with the electrode area *A*_Geo_ (0.194 cm^2^) and the Pt loading *L*_Pt_ (20 μg/0.196 cm^2^ ≈ 0.1 mg_Pt_ cm^−2^):
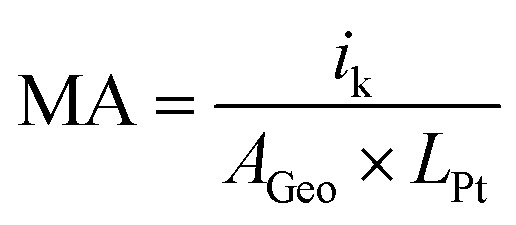


The ECSA was determined by integrating the charge in CO electrooxidation experiments between 0.4 V_RHE_ and 1.0 V_RHE_, following the approach reported in literature.^29,30^

### 2.3 Fabrication of membrane electrode assemblies

The novel PtCo/C catalyst was prepared by ReCatalyst. The commercial PtCo/C catalysts were purchased from Umicore N.V. 3M PFSA ionomer was supplied by 3M and PFSA FS715RFS membranes were supplied by Fumatech BWT GmbH.

Anode catalyst inks (2 wt% solids in 1 : 4 w/w IPA/water) were prepared using Pt/C (45 wt% Pt content, Elyst Pt50 0550, Umicore) and an ionomer-to-carbon ratio (I/C) of 0.7. Three cathode catalyst inks (2 wt% solids in 1 : 4 w/w IPA/water) were prepared using three different catalysts; PtCo/C (35 wt% Pt content provided by ReCatalyst). As references, two commercial PtCo/C from Umicore were chosen: Elyst Pt50 0690 (41 wt% Pt content) and Elyst Pt30 0690 (27 wt% Pt content), as these are two commercial PtCo/C catalysts reported in the literature with state-of-the-art performance^32–34^ and are available with comparable metal content to the ReCatalyst catalyst. The membrane electrode assemblies (MEA) with the PtCo/C from ReCatalyst are denoted ReCatalyst. The references MEAs with PtCo/C from Umicore are denoted UM50 for Elyst Pt50 0690 and UM30 for Elyst Pt30 0690. The ionomer-to-carbon (I/C) ratio was adjusted to 0.4 for all cathodes, based on previously published work from our group.^35^

The catalyst layers were applied onto pristine membranes using an automated ultrasonic spray-coating system (Sonaer Sono-Cell). Anode and cathode catalyst inks were applied onto commercial Fumapem® membrane (725 EW, mechanically reinforced, chemically stabilized, nominal thickness: 15 μm). The desired Pt-loading of all MEAs was 0.1 mg cm^−2^ for the anode and two different loadings for the cathode: 0.25 mg cm^−2^ and 0.4 mg cm^−2^. These loadings were chosen as 0.4 mg cm^−2^ is a standard Pt loading widely reported in literature and also present in typical commercial MEAs, while 0.25 mg cm^−2^ is a typical Pt loading envisioned for upcoming heavy-duty applications (*e.g.* summarized in the article of Cullen *et al.*).^36–40^ The loading was controlled by weighing a thin metal pad of 2 cm^2^ area before and after the spraying with a microbalance (ME36S, Satorius AG), as reported in a previous study.^41^ The resulting catalyst-coated membranes were sandwiched between two 4 cm^2^ gas diffusion layers (H14Cx653, Freudenberg). The performances of the 4 cm^2^ active area MEAs were evaluated using a fuel cell test station (Scribner 850e). All MEAs were tested with the same experimental protocol.

### 2.4 Characterization of membrane electrode assemblies

The protocol applied to all the MEAs in this work consisted of a break-in procedure followed by voltage recovery (VR). This procedure was shown to be a valuable step for removing sulfate, which improves the electrochemical performance.^32,42^ After the voltage recovery, polarization measurements were conducted in H_2_/O_2_, followed by cyclic voltammograms for the determination of electrochemical surface area (ECSA) and a hydrogen crossover measurement for a proper mass activity assessment. Lastly, polarization measurements in H_2_/air were performed.

The break-in procedure was reported in our previous work.^35^ The voltage recovery protocol is based on the works of Zhang *et al.*^43^ and Kabir *et al.*^32^ During the VR, the cells were held under 55 °C, above saturation (198% RH) and ambient pressure. A series of potential cycles between 0.08 V and 0.12 V was applied for 20 s each on the cells. The voltage cycles were repeated 180 times.

The H_2_/O_2_ polarization curves (0.25 slpm/1 slpm) were measured under 80 °C, 96% RH and 150 kPa_abs_. The current density was scanned from 0 mA cm^−2^ to 125 mA cm^−2^ in 5 mA cm^−2^ steps for 5 minutes per point (average of last 5 seconds used). High-frequency resistances (HFRs) were measured at a frequency of 3200 Hz by the fuel cell test station's integrated Frequency Resistance Analyzer (FRA) throughout all polarization characterizations and used to correct for membrane, contact, and electronic resistances, as previously reported in our work.^35^

The Tafel plots were corrected for the high-frequency resistances (HFR) and the hydrogen crossover current densities following the approach by Neyerlin *et al.*^44^ The mass activity is obtained by dividing the current density corrected with hydrogen crossover (*i* + *i*_x-over_) at 0.9 V_HFR-free_ with the cathode Pt-loading of the cell (0.25 mg_Pt_ cm^−2^).

The cyclic voltammograms (CVs) were performed under H_2_/N_2_, 35 °C, 95% RH and ambient pressure. The potential was swept from 0.05 to 1.0 V *versus* RHE at a scan rate of 50 mV s^−1^. The CVs were repeated 8 times to reach saturation. The cyclic voltammograms of the three samples are provided in the ESI.† We acknowledge that the hydrogen underpotential deposition (H_upd_) method for Pt-alloy complicates the quantitative ECSA measurement due to the altered electronic properties of PtCo, as this change affects the adsorption behaviour of hydrogen.^45,46^ Still, the H_upd_ method is widely used as a standard in literature for both Pt^47^ and Pt-alloy catalysts^48,49^ and is therefore employed in this work. As reported in the literature, the factor used to calculate the ECSA from the H_upd_ charge was 210 μC cm^−2^.^32,35^ The more accurate values for the ECSA are obtained by CO-stripping (see section 2.2.5), as the adsorption of CO is less affected by the altered electronic properties of PtCo.^46^ However, differences in ECSAs will be discussed in section 3.2.1.

Hydrogen crossover currents were measured *via* linear sweep voltammetry (LSV) under H_2_/N_2_ and under the same conditions as the H_2_/O_2_ polarization curves, *i.e.* 80 °C, 96% RH and 150 kPa_abs,_ to correct the current densities from the H_2_/O_2_ polarization curves.

The H_2_/air polarization curves (0.25 slpm/0.5 slpm) were measured at 80 °C, 96% RH and ambient pressure. This condition ensures no additional effects on the water management induced by the backpressure or the proton conductivity caused by the reduced gas humidity, providing a simple comparison between the catalysts on the MEA level.

The current density was scanned from zero to 250 mA cm^−2^ in 12.5 mA cm^−2^ increments with a 1 minute hold at each current step.^35^ Full polarization curves were obtained by scanning the current density from 375 mA cm^−2^ to the final current density in 125 mA cm^−2^ increments with a 3 minute hold per current step.^35^

## 3 Results and discussion

### 3.1 Particle composition and morphology

**Fig. 1:**
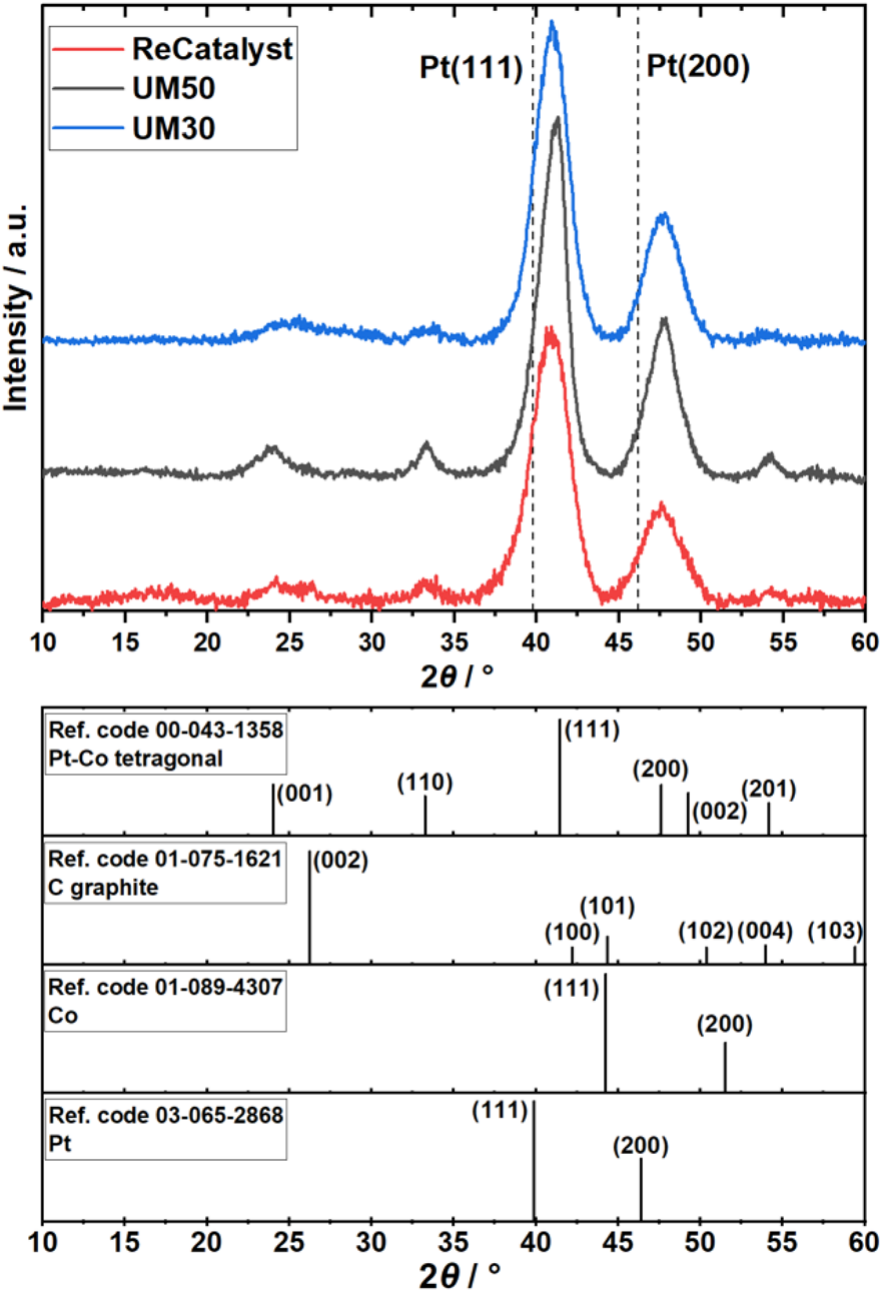
Top: X-ray diffraction patterns of the ReCatalyst (PtCo/C synthesized *via* galvanic displacement, red) and the two benchmark catalysts Umicore UM30 (blue) and UM50 (black). Bottom: Line patterns of reference materials and their commonly assigned crystal planes.

ICP-OES measurements revealed the Pt:Co compositions of the three catalysts to be Pt_2.8_Co for Umicore Elyst Pt30 0690 (UM30), Pt_2.4_Co for Elyst Pt50 0690 (UM50) and Pt_2.9_Co for PtCo/C synthesized *via* double-passivation galvanic displacement (ReCatalyst). XRD patterns of all three samples (Fig. 1) confirm the high-crystallinity and bimetallic alloy nature of all PtCo/C catalysts, whereby both are also evidenced by aberration-corrected STEM and STEM-EDX element mapping (Fig. S1 and S2†), respectively. The diffractograms suggest the close similarity of the bulk chemical composition as well as the crystal structure of the as-prepared ReCatalyst and PtCo/C references from Umicore. The diffraction peaks at 2*θ* = 24–26°, 33–34°, 40.9–41.3°, 47.6–47.9° and 54° correspond to the (001), (110), (111), (200) and (201) planes of intermetallic tetragonal *P*4/*mmm* PtCo. The peak at 2*θ* = 24–26° is superimposed by the (002) plane of the carbon support.^50^ Compared to standard *face-centered cubic* (fcc) platinum, the dominant (111) and (200) plane peaks (denoted by the dashed lines, PDF(Pt)#00-004-0802) are shifted to higher 2*θ* values, indicating a substantial lattice contraction due to the formation of the alloy. Employing Bragg's law, interplanar spacings of 2.25 Å (UM30), 2.20 Å (UM50), and 2.21 Å (ReCatalyst) for the (111) plane were obtained.^51^ These values are approximately in agreement with d-spacings of PtCo alloys reported in the literature and are slightly shortened compared to the *d*-spacings reported for pure platinum nanoparticles (∼2.3 Å).^52–56^

**Fig. 2:**
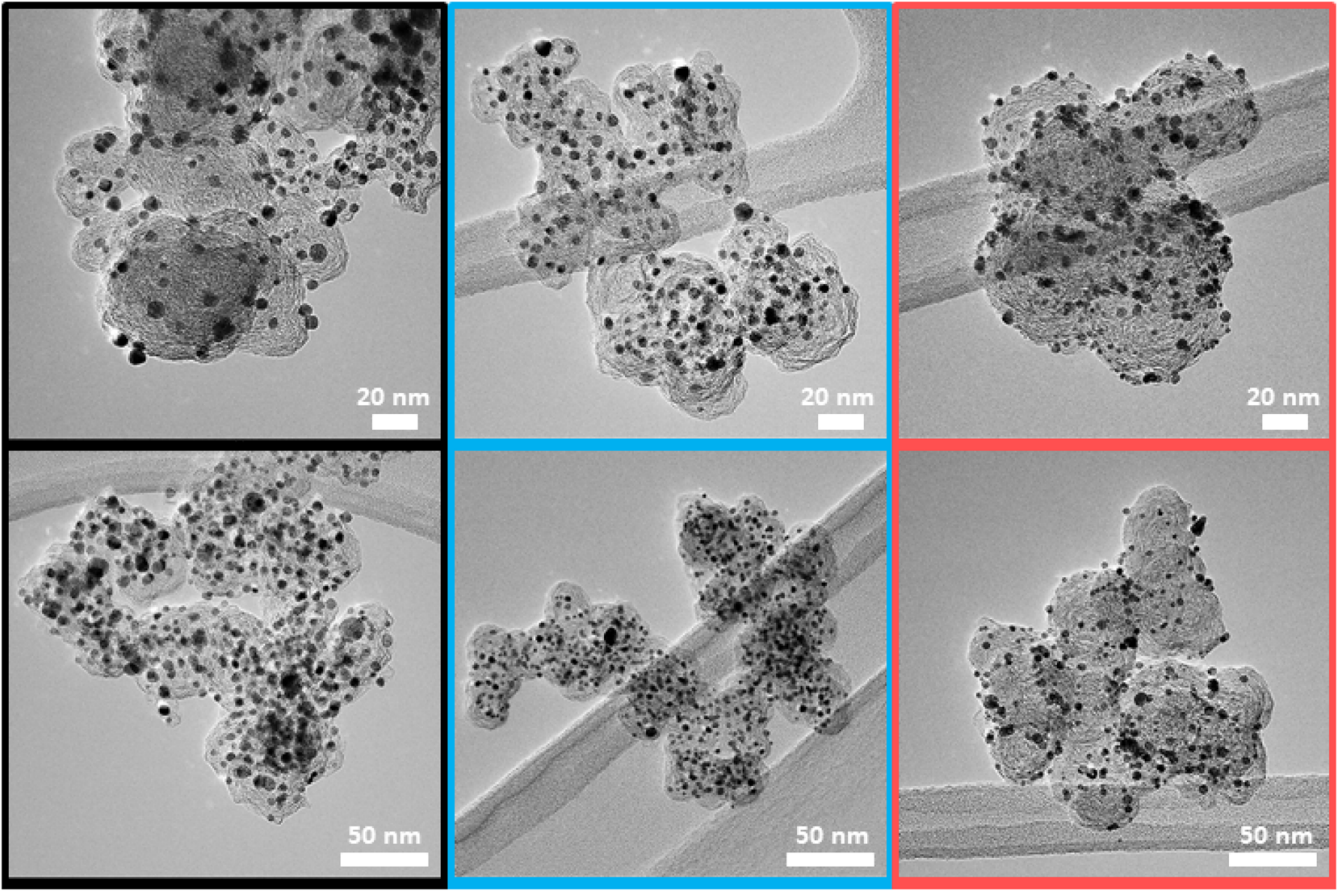
Representative TEM micrographs of the three catalysts UM50 (black), UM30 (blue) and ReCatalyst (red).

The observed lattice distance decrease could result from the incorporation of the smaller cobalt atoms (atomic radius of 1.26 Å) in place of the larger platinum atoms (1.36 Å).^57,58^ The pronounced broadening of these reflections in all three samples suggests a small mean crystallite size. As the width of a diffraction peak is not only influenced by the crystallite size, but also by crystal lattice imperfections such as dislocations, chemical inhomogeneities and residual stress, the simple measurement of the full width at half maximum (FWHM) to calculate specific crystallite size values (*e.g.* by the often used Scherrer equation^59^) can be biased by the mentioned effects.^60^ Still, a decreasing FWHM was observed in order of UM50 (2.0°) < UM30 (2.4°) < ReCatalyst (2.7°) for the (111) plane, suggesting a slightly larger crystallite size for the Umicore references. To obtain more precise information about the particle morphology, additional TEM micrographs over a larger field of view were acquired (Fig. 2, more micrographs can be found in Fig. S3–S5†). The carbon support in all three samples consists of 30–80 nm primary carbon particles, which coalesce into aggregates. These primary carbon particles are characterized by core-filling amorphous and shell-like graphitic carbon (see more resolved HRTEM micrographs in Fig. S6†). The resemblance of the carbon support in all samples, as well as the similarity of the XRD patterns and former findings in their electrochemical behaviour confirms that the Umicore references also contain a high surface area carbon as support material.^25^ It can be seen that on all samples, the PtCo nanoparticles are finely distributed over the entire carbon surface. From 2D TEM micrographs it is not possible to determine the precise proportion of nanoparticles located inside or outside the carbon pores. As proposed by Harzer *et al.*, however, some nanoparticles can be reliably assigned to be on the outside of the carbon support if the nanoparticle is clearly visible outside the projection of the carbon primary particle. In turn, it cannot be resolved whether a particle sits on the surface or in a carbon pore if that nanoparticle is completely enclosed by the projection of the primary carbon particle.^61^

With this counting method, it can be estimated that more nanoparticles are located on the carbon surface for the ReCatalyst compared to the Umicore catalysts, as notably more particles are located outside the carbon projections. This is supported by TEM tilt series (ESI movies 1–3†), where the rotation of the respective carbon particles over a wide tilt range (typically 140–144°) allows a better determination of the nanoparticle position.

**Fig. 3:**
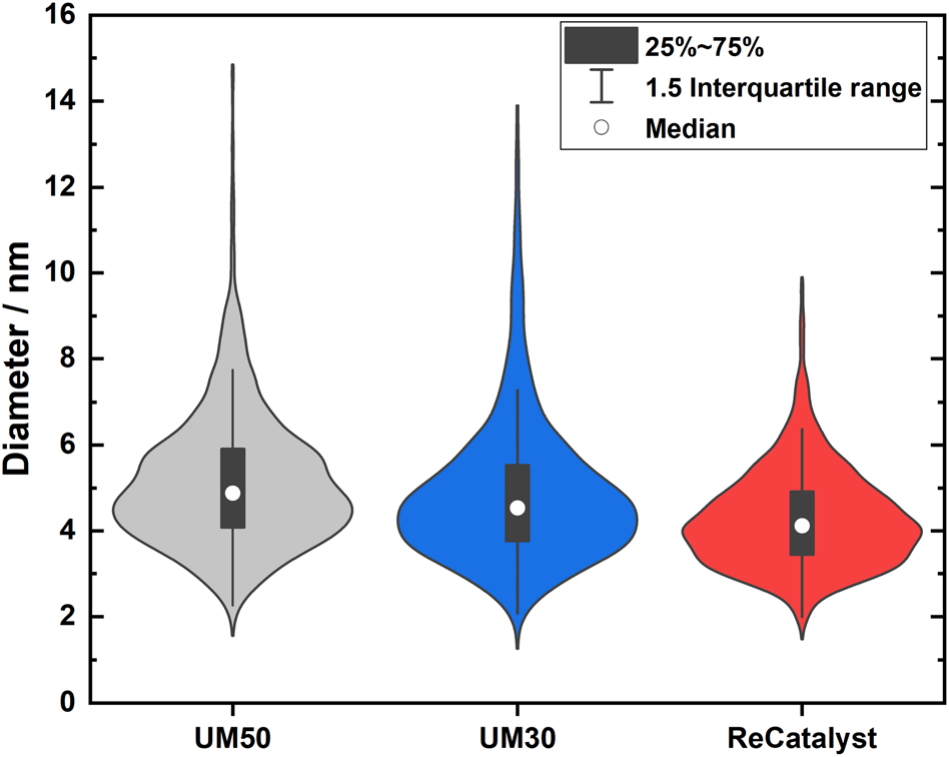
Violin plots of the particle size distribution for the three PtCo/C catalysts.

One of the main advantages of the developed alternative synthesis route is the ability to obtain a narrower particle size distribution, which in general could also be considered as advantageous towards the reduction of excessive Ostwald ripening of the particles upon aging.^62^Fig. 3 shows a violin plot with the particle size distribution obtained by measuring the diameters of the nanoparticles in the 2D TEM micrographs to enable a facile comparison of the PSDs of all three catalysts.

**Fig. 4:**
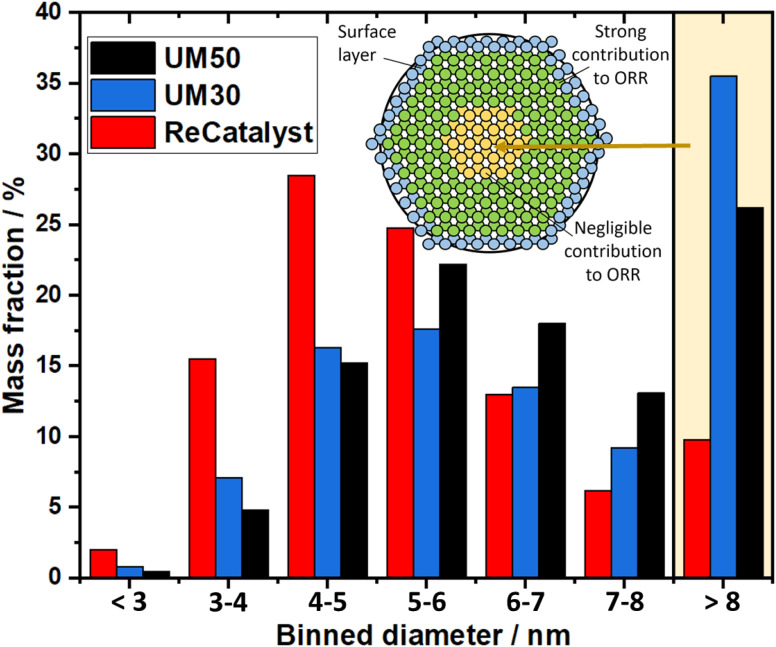
Mass distribution of the three PtCo/C catalysts.

The vast majority of the measured nanoparticles for all three samples feature a diameter of 3 to 6 nm. The number-weighted diameter was determined to be 4.8 nm for UM50, 4.5 nm for UM30 and 4.1 nm for ReCatalyst, while the surface normalized diameters were calculated to be 6.3 nm for UM50, 6.1 nm for UM30 and 5.1 nm for ReCatalyst, respectively.^61^ In comparison, a diameter of ∼4.4 nm was reported for the Umicore Elyst Pt 50 0670 variant.^63,64^ Following Schulenburgs' *et al.* approach for more realistic determinations of the surface area by TEM micrograph evaluation, we approximated the TEM derived surface areas of the samples to be 68 m^2^ g_Pt_^−1^ for UM50, 75 m^2^ g_Pt_^−1^ for UM30 and 86 m^2^ g_Pt_^−1^ for ReCatalyst assuming spherical particles.^45^ It is important to note that although the majority of nanoparticles have comparable diameters, it is the significantly smaller number of particularly large (>8 nm) nanoparticles in the ReCatalyst sample that plays a crucial role in the utilization of the employed precious metal, which is directly quantified by the ECSA (see Table 1). While the surface area of the ReCatalyst catalyst from purely geometric considerations is already 26% and 17% higher compared to UM30 and UM50, respectively, these differences are substantially greater for the electrochemical surface area and are discussed in section 3.3. Both size and geometry are fundamentally determinating the active sites of the particles (besides the chemical composition) and thus influence the critical selectivity and stability of the catalyst.^65^ This is called the particle size effect where smaller particles exhibited lower specific ORR activity and lower stability.^66–68^ To not underutilize precious metal in the bulk of the particle and risk mass activity losses, it is essential to ensure large enough diameters that allow electrochemical activity and sufficient stability of the particles, but at the same time show a high surface-to-volume ratio for electrochemical surface maximization. These relationships have been extensively modelled and experimentally investigated for the simpler Pt/C system, where Pt nanoparticles with diameters of ∼2–4 nm have been determined to feature optimal mass activity.^69–72^ Even though particle size studies of PtCo nanoparticles are not similarly extensive and are more difficult to obtain due to the alloy nature of nanoparticles with varying intraparticle elemental (*e.g.* Pt/Co ratio) and structural (*e.g.* degree of ordering) compositions, similar trends as in pure Pt-particles seem to apply.^19,73,74^*E.g.* according to Wang *et al.* the maximum mass activity for Pt_3_Co nanoparticles is found at particle sizes of ∼4.5 nm, although it must be pointed out that this activity also depends strongly on numerous other parameters (*e.g.* element composition, particle shape).^75,76^ In Fig. 4, the highest mass fraction for ReCatalyst is found at 4–5 nm, which lies in this optimal range for catalyst activity. For the commercial catalysts, the distribution is slightly shifted to 5–6 nm. This trend is in line with the improvement in RDE derived ECSA and mass activity of the ReCatalyst material compared to the UM references (see below, Fig. S7 and S8 and Table S1†).

**Table 1:** Electrochemical surface area of three catalysts obtained by H_upd_ in MEA setup with two different Pt-loadings (0.4 mg cm^−2^ and 0.25 mg cm^−2^) and CO-stripping with thin-film RDE. TEM measurements determined the estimated available surface

Sample	Pt loading in mg cm^−2^	CL thickness in μm	ECSA in m^2^ g^−1^ H_upd_	Roughness factor in cm_Pt_^2^ cm_MEA_^−2^ (with ECSA from H_upd_)	ECSA in m^2^ g^−1^ TEM	ECSA in m^2^ g^−1^ TF-RDE
UM50	0.4	8	31	124	68	59
	0.25	5	23	58		
ReCatalyst	0.4	17	38	152	86	96
	0.25	10	45	113		
UM30	0.4	32	37	148	75	70
	0.25	18	43	108		

A second important factor influencing the catalyst performance is the loss of active catalyst material in large catalyst particles: For assumed solid spherical particles with a diameter of 6 nm or more, more material is found inside the particle than at the surface marking the lower limit above which the proportion of inside and outside material inflects, due to spheres having the lowest surface to volume ratio. In nature, nanoparticles are not perfectly spherical and not only the first atomic surface layer but also the subsequent atomic layers below have a crucial influence on the reaction, as shown with DFT calculations by Patrick *et al.*, albeit with decreasing significance.^14^ It can be expected that above a particle size of ∼8 nm the majority of the core atoms play a negligible role in the oxygen reduction reaction and thus the contribution of the fractional mass activity of these particles to the total mass activity of the catalyst is almost zero. Applying this consideration to the analysis of our TEM investigation summarized in the binned histogram in Fig. 4 (binned values in Table S2†), we find a significantly reduced mass ratio of particles >8 nm for the ReCatalyst (∼10%) *vs.* the Umicore references (∼26% and 35% for Umicore 50 and 30, respectively), a consequence of the strong weighting of large particles due to the cubic relationship between the diameter and volume of spheres and, consequently, the mass of PtCo particles. This finding clearly confirms the advantage of the alternative fabrication procedure given the sharper particle size distribution for the ReCatalyst material.^23–25^ Nevertheless, one can assume considerable benefits are still accessible with further optimization of the particle size distribution if a complete elimination of particles with diameters >8 nm is achieved. The mass fraction of particles <3 nm is very low in all three samples (below ∼2.5% for all samples). Since very small particles feature a strong curvature, they are prone to faster decomposition due to increased surface energy and are therefore unfavourable for catalyst long-term durability.^65,77^ We also acknowledge, however, that the detection of nanoparticles in the sub-nm range is difficult due to the acquisition of TEM micrographs with an intermediate field of view (typically 50–200 nm) with associated reduced pixel resolution, which could be circumvented by improved imaging equipment in future work.

### 3.2 Electrochemical performance

Given the different size distributions of the three catalysts, the following sections show the concomitant ECSA (section 3.2.1) and the samples' electrochemical performance at the MEA level (section 3.2.2).

#### 3.2.1 Electrochemical surface area

Table 1 shows the ECSA of the three catalysts obtained by different methods: H_upd_ in MEA setup, CO-stripping with TF-RDE and TEM measurements. The ECSAs of all samples measured with CO-stripping in the thin-film rotating disk electrode (TF-RDE) setup are at least 1.9 times higher than those obtained with H_upd_ in the MEA setup, similar to the results reported in the literature.^30,46,49^ We are aware that the H_upd_ for Pt-alloy have to be interpreted with care, since the altered electronic properties of PtCo may affect the adsorption behaviours of hydrogen more than of CO. Nevertheless, the qualitative comparison between the three samples confirms the observed trend in ECSA values, measured by TEM and TF-RDE.

At the same Pt loading on the cathode as both catalyst powder and as an MEA and independent from the measurement technique (MEA, TEM, RF-RDE), the ECSA of ReCatalyst is the highest compared to those of the reference samples, especially compared to that of UM50, *i.e.* ReCatalyst has the highest roughness factor, which is the product of the ECSA and the CL loading.^78^ The higher roughness factor of the ReCatalyst PtCo/C correlates well with its narrower particle size distribution, especially compared to UM50 (Fig. 3).

Theoretically, the ECSA should not be affected by the Pt loading of the cathode CL. However, the ECSA was practically affected by the loading, which is related to other properties, *e.g.* the CL thickness and utilization. For instance, the ECSA of the CL with UM50 increased significantly (by 35%) with higher loading (and thickness), while a reverse trend was observed for ReCatalyst and UM30: the ECSA decreased (by less than 20%) with higher loading. It is important to note that the thickness of the CL with UM50 is increased to less than 10 μm (from 0.25 mg_Pt_ cm^−2^ to 0.4 mg_Pt_ cm^−2^), while the CL thicknesses were greater than 17 μm for ReCatalyst with 0.4 mg_Pt_ cm^−2^ and UM30 with both loadings (Table 1 and Fig. S12†). Based on these observations, CL thicknesses higher than 10 μm could have detrimental impacts on the ECSA of the MEAs. Further information obtained from the *in situ* CVs is shown in Fig. S9.†

#### 3.2.2 *In situ* electrochemical performance

At 0.4 mg_Pt_ cm^−2^, UM50 PtCo/C showed the highest mass activity (138 A g_Pt_^−1^), while at 0.25 mg_Pt_ cm^−2^, the mass activity of UM30 was the highest (173 A g_Pt_^−1^) compared to the two other catalysts despite the lower ECSAs of UM50 and UM30 PtCo/C compared to that of ReCatalyst. Like the ECSA, the mass activity of a catalyst should theoretically not depend on the loading of the catalyst layer. While UM50 PtCo/C shows similar mass activities (∼138 A g^−1^) at the two given Pt loadings and the concomitant CL thicknesses, the mass activity of UM30 PtCo/C increased by 43% with the reduced Pt loading (0.4 mg cm^−2^ to 0.25 mg cm^−2^) and CL thickness (from 32 μm to 18 μm). The mass activity of ReCatalyst PtCo/C also increased by 39% with the reduced CL thicknesses (17 μm to 10 μm), indicating the impact of CL thickness on the mass activity of a catalyst.

At similar thicknesses (8–10 μm), ReCatalyst PtCo/C has a higher mass activity (146 A g^−1^) than that of UM50 (138 A g^−1^). This trend aligns with the ECSA measurements and TF-RDE (ESI Table S1†) for the two catalysts. The higher mass activity can be attributed to the narrower particle size distribution of ReCatalyst especially compared to UM50 (Fig. 3). However, also at similar CL thicknesses (17–18 μm), ReCatalyst PtCo/C has a lower mass activity (105 A g^−1^) than that of UM30 PtCo/C (173 A g^−1^). The trend in mass activity is again in agreement with that of the ECSA_Hupd_ (ReCatalyst < UM30) (Table 2), however, it does not match the results obtained by TF-RDE (Table S1†), and direct comparisons between H_upd_ derived ECSAs and TF-RDE derived ECSAs should be made with caution for the reasons stated above.

**Table 2:** Mass activities of the three catalysts determined with *in situ* characterization under H_2_/O_2_, 80 °C, 96% RH, 150 kPa_abs_ at different loadings: 0.4 and 0.25 mg_Pt_ cm^−2^

Sample	Pt loading in mg cm^−2^	CL thickness in μm	Mass activity in A g^−1^	ECSA (H_upd_) in m^2^ g^−1^
UM50	0.4	8	138	31
	0.25	5	137	23
ReCatalyst	0.4	17	105	38
	0.25	10	146	45
UM30	0.4	32	121	37
	0.25	18	173	43

The mass activity of ReCatalyst PtCo/C is slightly lower than that of UM30 in MEA configuration. This might be linked to the fact that ReCatalyst PtCo/C features a higher fraction of exterior PtCo on carbon than the references (as discussed earlier), which increases the chance for ionomer-induced poisoning for ReCatalyst catalyst *vs.* UM30 and was reported to lower mass activity.^79–82^ Further, the lower PtCo content in UM30 (with a lower fraction of exterior PtCo on carbon) might help to reduce the exposure of the catalyst to ionomer.^83^

**Fig. 5:**
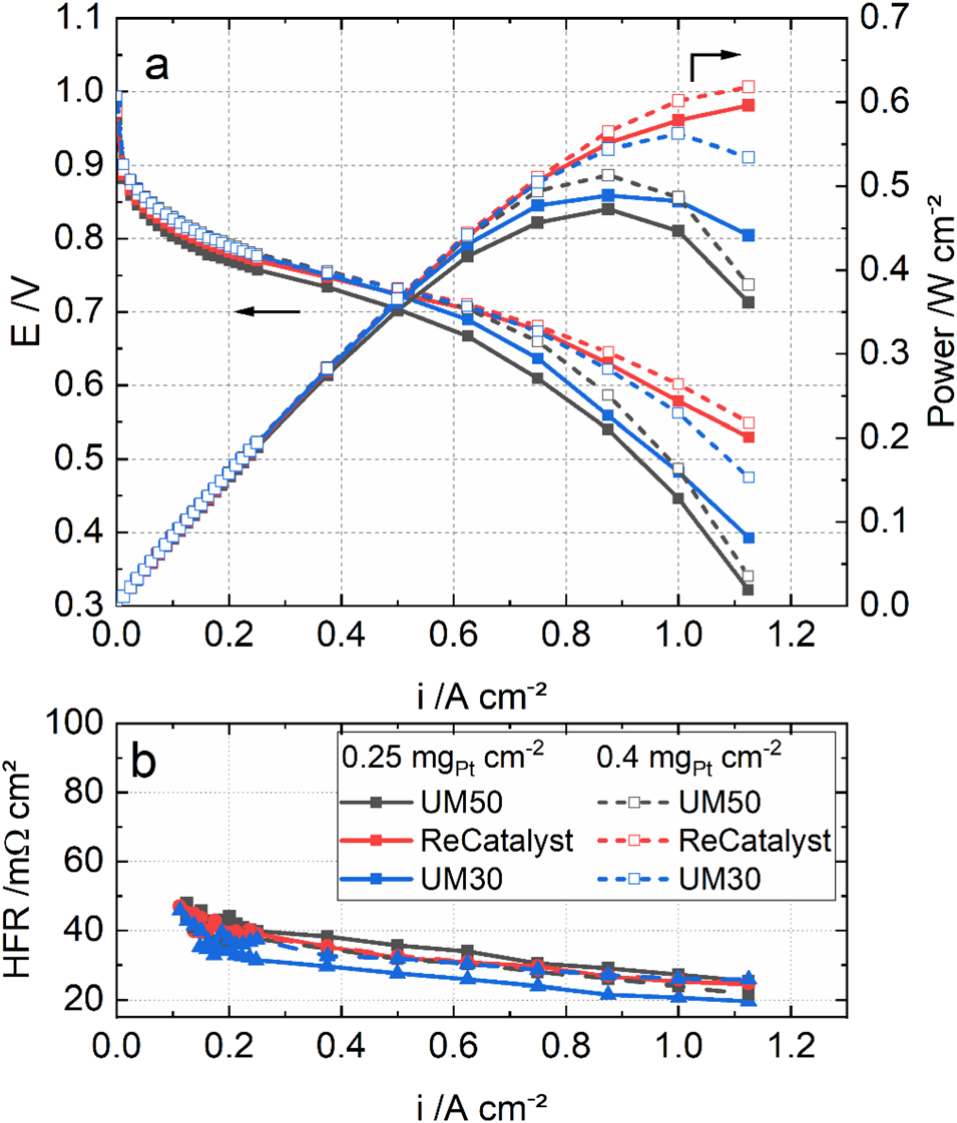
Polarization curves (a) and the high-frequency resistances (b) of the MEAs with UM50, ReCatalyst and UM30 at 0.25 mg_Pt_ cm^−2^ loading and 0.4 mg_Pt_ cm^−2^ loading under H_2_/air, 80 °C, 96% RH and ambient pressure.

Fig. 5 shows the polarization curves of the MEAs with the three catalysts under H_2_/air, 80 °C, 96% RH and ambient pressure. In particular, at high current densities (>750 mA cm^−2^), the MEA performance with ReCatalyst is superior at the same Pt loadings. This result is most likely linked to the significantly higher roughness factor of ReCatalyst (Table 1) and probably also to a higher fraction of exterior PtCo particles on carbon (as discussed earlier).

Fig. 5 also compares the MEAs with the three catalysts in two different cathode loadings: 0.4 mg_Pt_ cm^−2^ and 0.25 mg_Pt_ cm^−2^. It can be seen that the performance of the ReCatalyst MEA is not affected much by the reduced loading. The MEA with 0.25 mg_Pt_ cm^−2^ ReCatalyst PtCo/C still outperforms that with 0.4 mg_Pt_ cm^−2^ UM50 in both conditions. This result indicates that the higher peak performance of the ReCatalyst MEA (0.25 mg_Pt_ cm^−2^) is mainly attributed to the greater ECSA and the related roughness factor in combination with a possibly higher fraction of exterior PtCo on carbon than the references. These factors improve the oxygen assessment to the Pt surface at high current densities.^4,78^ Further, as the catalyst layer thicknesses of UM50 at 0.4 mg_Pt_ cm^−2^ and ReCatalyst at 0.25 mg_Pt_ cm^−2^ are both approx. 10 μm (Table 1), the performance improvement at high current densities can be considered independent from the catalyst layer thickness. This is reflected in the cell metrics: The performance of the ReCatalyst-MEA was reduced only by 4% at 0.7 V and peak power density (PPD) when the Pt-loading was reduced from 0.4 to 0.25 mg_Pt_ cm^−2^. The performance at 0.7 V was reduced by 20%, while the peak power density was reduced by 8% with UM50. The performance at 0.7 V was reduced by 10% and 14% at PPD with UM30.

## 4 Conclusion and outlook

Based on an alternative catalyst synthesis approach *via* the double passivation galvanic displacement, we have shown that a significant improvement in the particle size distribution can be obtained compared to commercial benchmark PtCo catalysts. TEM and XRD characterization confirmed that our optimized ReCatalyst PtCo/C catalyst features a considerably lower mass fraction of particles >8 nm *vs.* the commercial references. In addition, higher fractions of nanoparticles are located on the carbon surface for the ReCatalyst compared to the Umicore catalysts. These two features enable more effective usage of the available active catalyst material, which is reflected in superior single-cell performance in particular at reduced Pt-loading of 0.25 mg cm^−2^ at the cathode. Future works should further optimize this new catalyst synthesis and MEAs preparation, also in relation to an extended range of cathode loadings (*e.g.* low-loaded cathodes with <0.1 mg cm^−2^ Pt loading for light-duty applications). In particular, screening different carbon support pore sizes and closing the herein observed gap between RDE and MEA mass activity values to finally exploit the full potential of those novel catalysts. In further studies, the long-term stabilities of the electrocatalysts and its contribution to the overall MEA durability would also be of vital research importance.

## Conflicts of interest

The authors declare no competing financial interest.

## Supplementary Material

RA-013-D2RA07780A-s001

RA-013-D2RA07780A-s002

RA-013-D2RA07780A-s003

RA-013-D2RA07780A-s004
