# Supplementary material for: Alternative and facile production pathway towards obtaining high surface area PtCo/C intermetallic catalysts for improved PEM fuel cell performance
Source: RSC Adv. 2023 Feb 6;13(7):4601–11. doi: 10.1039/d2ra07780a (PMC9900476; doi:10.1039/d2ra07780a)
Supplement: RA-013-D2RA07780A-s001 [file RA-013-D2RA07780A-s001.pdf]

Electronic Supplementary Material (ESI) for RSC Advances.
This journal is © The Royal Society of Chemistry 2023

Supporting Information

Alternative and facile production pathway towards obtaining high surface area PtCo/C intermetallic catalysts for improved PEM fuel cell performance

Philipp A. Heizmann[1,2,=], Hien Nguyen[1,3,=], Miriam von Holst[1,3], Andreas Fischbach[1], Mitja Kostelec[4], Francisco Javier Gonzalez Lopez[4,5], Marjan Bele[4], Luka Pavko[4], Tina Đukić[4], Martin Šala[6], Francisco Ruiz-Zepeda[4], Carolin Klose[1,3], Matija Gatalo[4,5], Nejc Hodnik[4], Severin Vierrath[1,2,3] and Matthias Breitwieser[1,3,z]

1. Electrochemical Energy Systems, IMTEK - Department of Microsystems Engineering, University of Freiburg, Georges-Koehler-Allee 103, 79110 Freiburg, Germany
2. University of Freiburg, Institute and FIT – Freiburg Center for Interactive Materials and Bioinspired Technologies, Georges-Köhler-Allee 105, 79110 Freiburg, Germany
3. Hahn-Schickard, Georges-Koehler-Allee 103, 79110 Freiburg, Germany
4. Department of Materials Chemistry, National Institute of Chemistry, Hajdrihova ulica 19, 1000 Ljubljana, Slovenia
5. ReCatalyst d.o.o., Hajdrihova ulica 19, Ljubljana, 1000, Slovenia
6. Department of Analytical Chemistry, National Institute of Chemistry, Hajdrihova ulica 19, 1000 Ljubljana, Slovenia

= Equal contribution

z Corresponding author. E-Mail: matthias.breitwieser@hahn-schickard.de

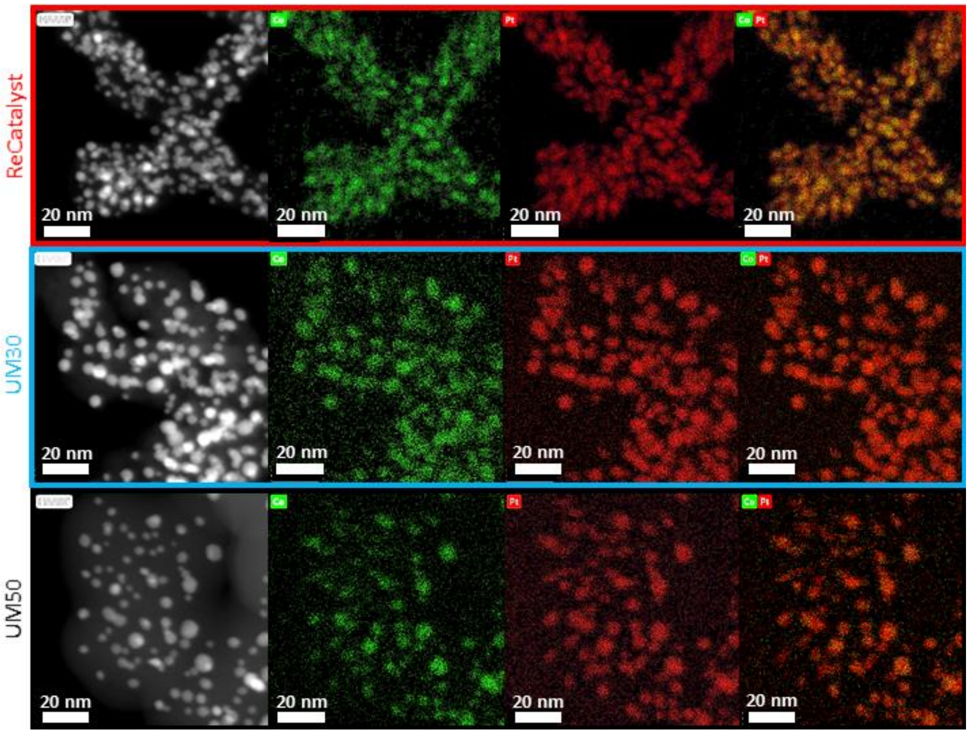

Figure S1: STEM-EDX Mapping (red: Pt, green: Co) of the three PtCo/C electrocatalysts showing the alloy nature of the PtCo nanoparticles: ReCatalyst (always in red), UM30 (always in blue) and UM50 (always in black).

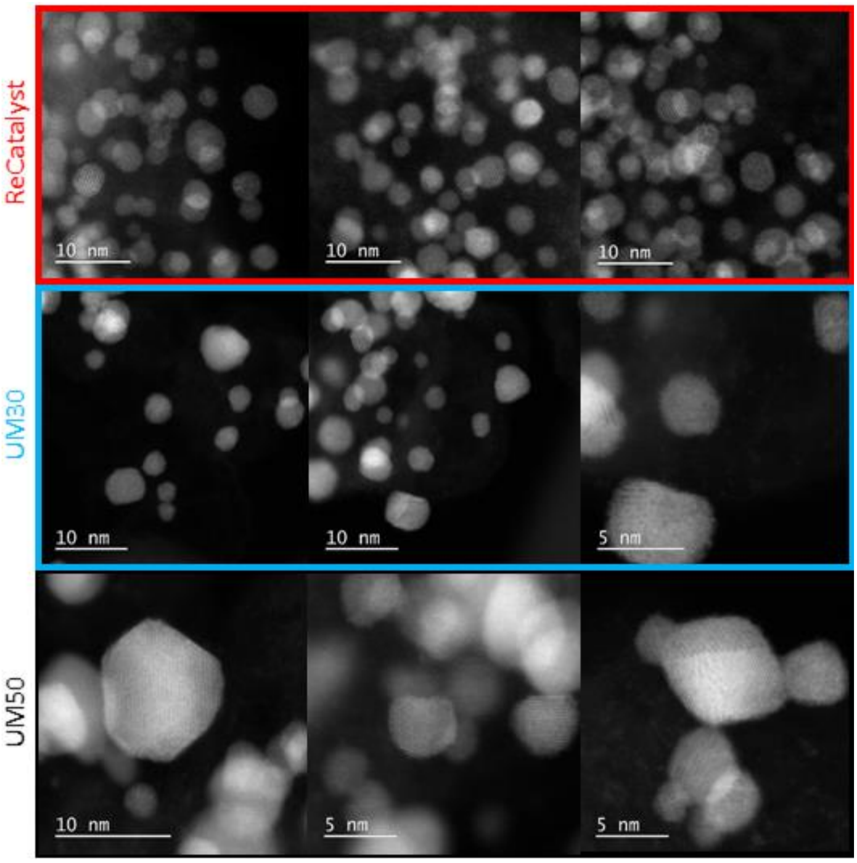

Figure S2: Aberration-corrected STEM micrographs showing the high crystallinity of the nanoparticles for all three samples.

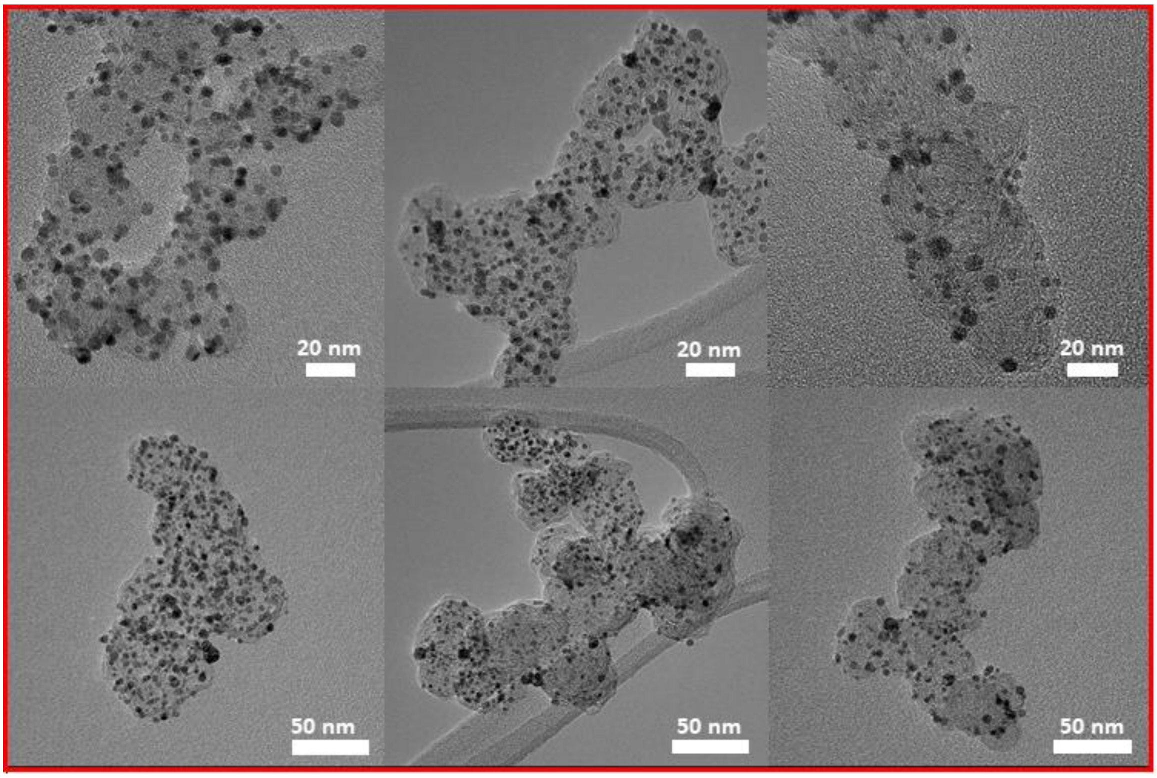

Figure S3: Additional TEM micrographs of the ReCatalyst sample.

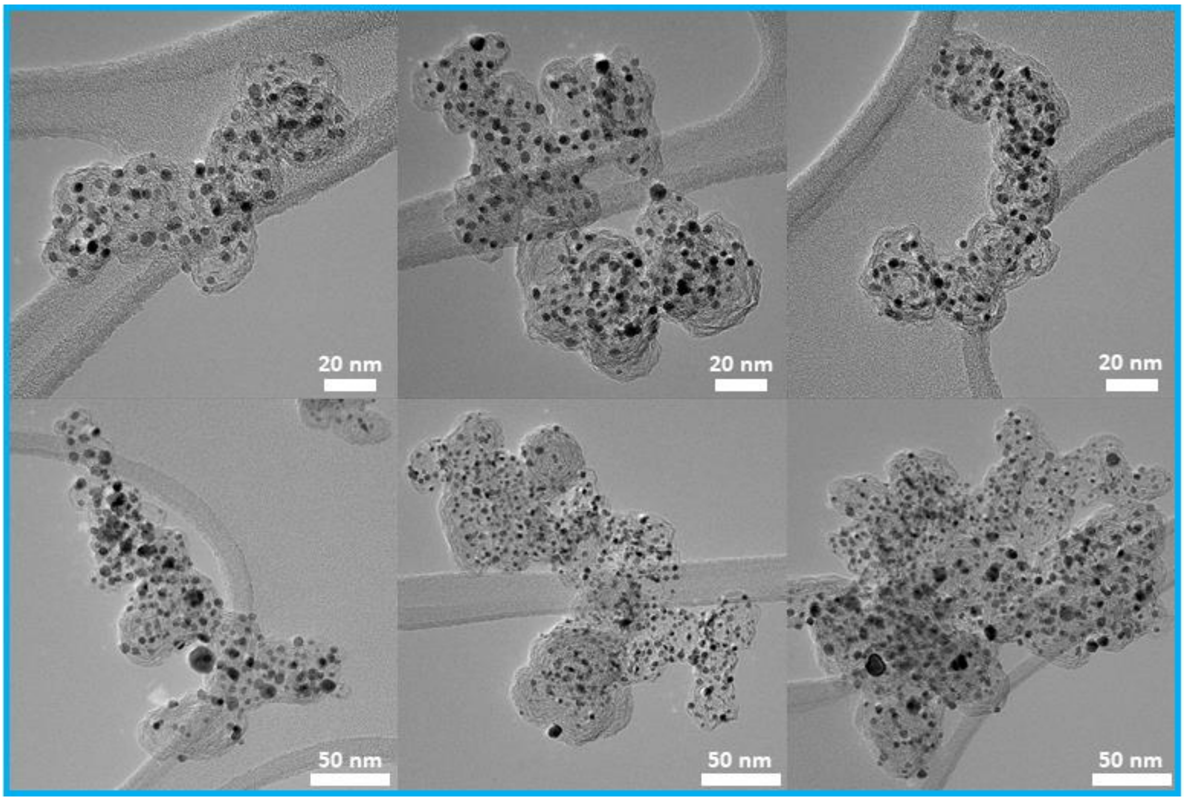

Figure S4: Additional TEM micrographs of the UM30 sample.

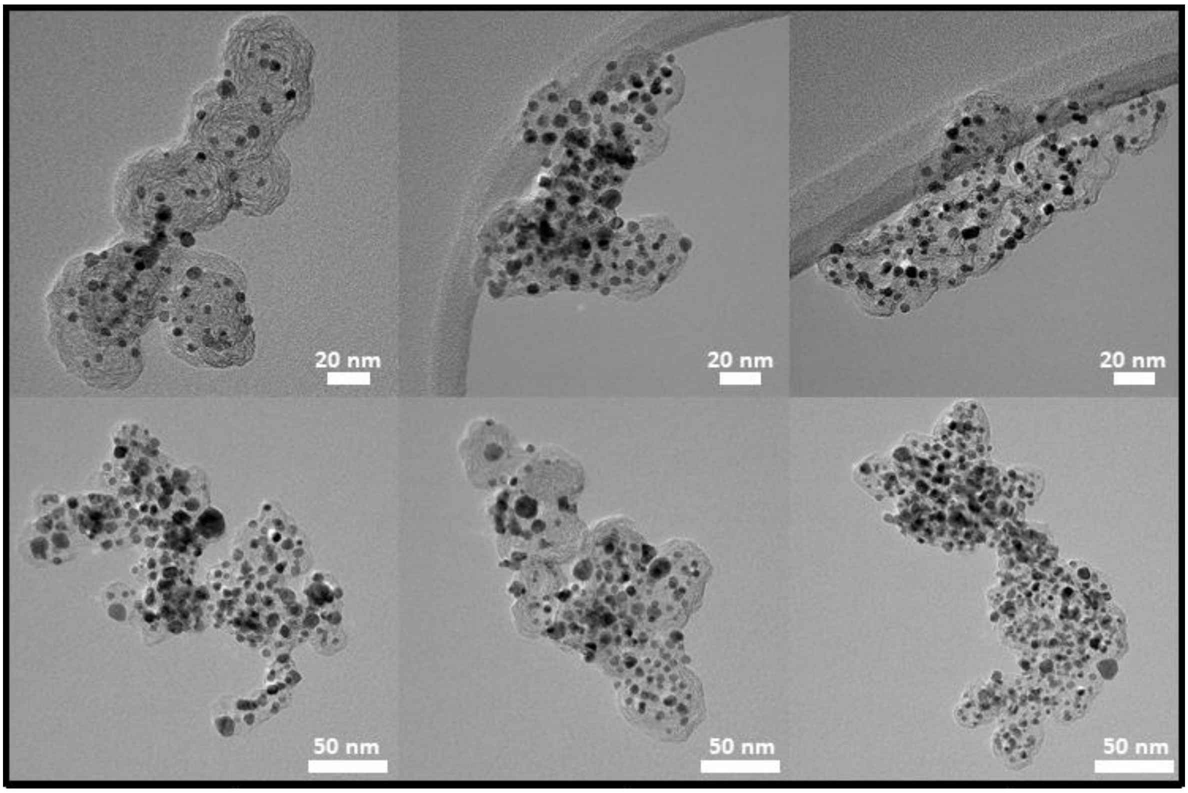

Figure S5*:* Additional TEM micrographs of the UM50 sample.

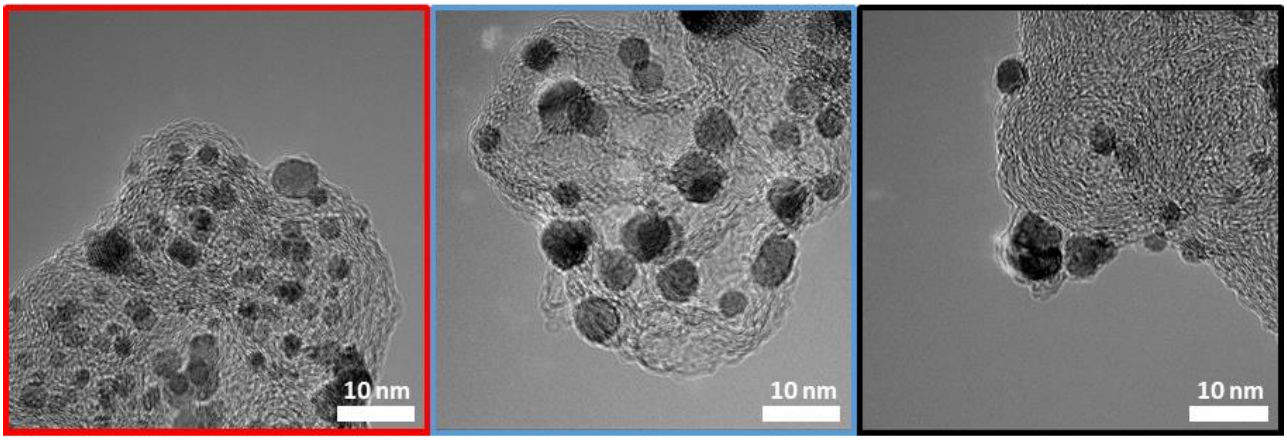

Figure S6: Additional HRTEM micrographs of the carbon support: ReCatalyst (red), UM30 (blue) and UM50 (black).

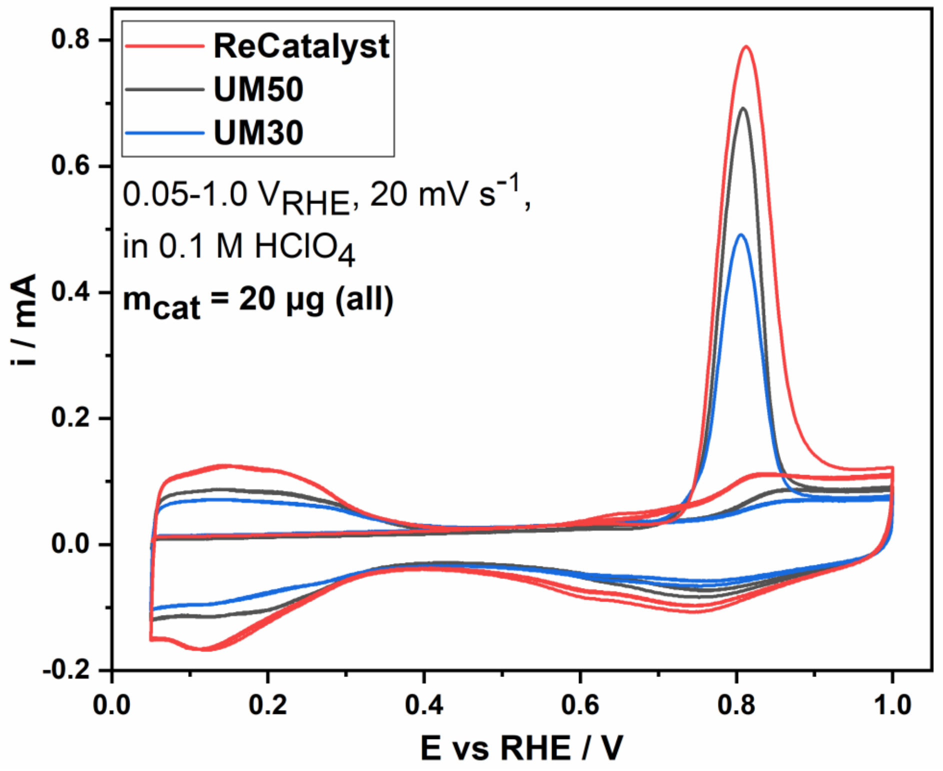

Figure S7*:* CO-electrooxidation cyclic voltammograms of the electrocatalysts obtained from TF-RDE.

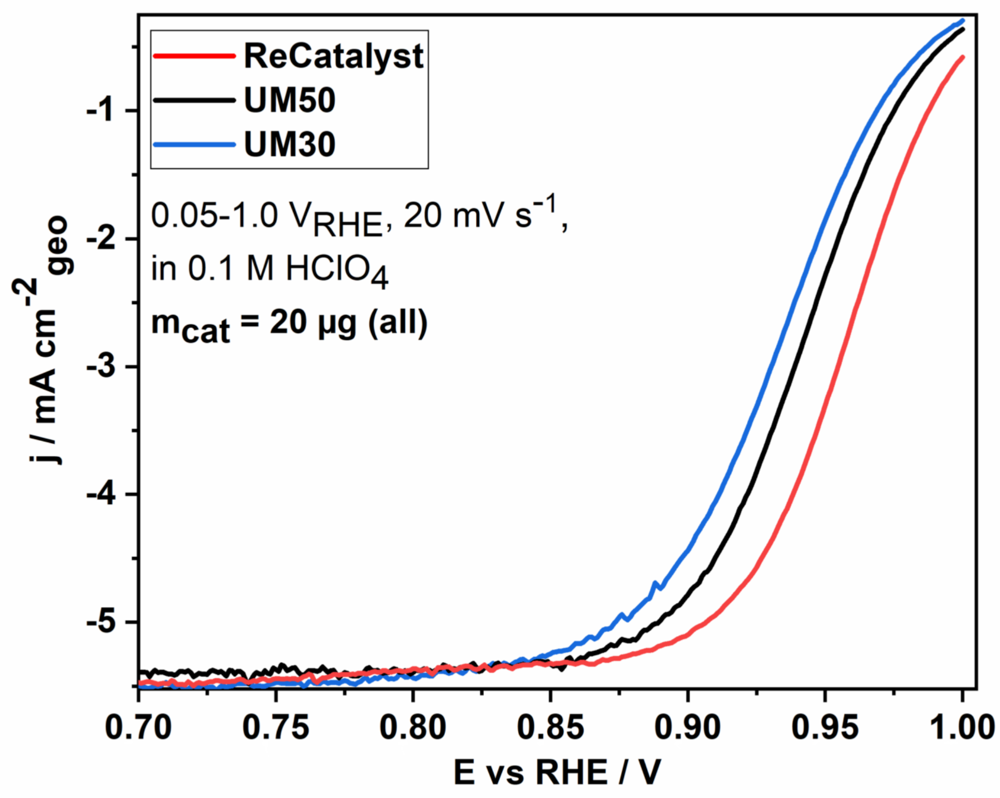

Figure S8: ORR polarization curves of the electrocatalysts obtained from TF-RDE.

Cyclic voltammograms (H_2/N_2)

Figure S show the cyclic voltammograms and the obtained electrochemical surface areas (ECSA) of the three electrocatalysts. The different size distribution and the concominant different in interparticle distance (shown in previous section) has been shown to affect the ECSA and catalytic activity.[42, 43]

It shows that the current density in the hydrogen adsorption/desorption potential region of the ReCatalyst catalyst is similar to that of UM30, however, the capacitive current density of the MEA with ReCatalyst Pt-Co/C is closer that of UM50 than to that of UM30.

The total capacity C_{dl} of the electrode is accounted to the ECSA (Pt area) and the carbon area (s. Equation 1).[44]

$$C_{\mathrm{dl}} = A_{\mathrm{Pt}} \cdot C^{*}_{\mathrm{dl,Pt}} + A_{\mathrm{Carbon}} \cdot C^{*}_{\mathrm{dl,Carbon}} \quad (1)$$

where $C^{*}_{\mathrm{dl,Pt}}$ and $C^{*}_{\mathrm{dl,Carbon}}$ are the specific double layer capacitance (in F cm^{-2}) for Pt and carbon, respectively.[44] A_{Pt} (ECSA x Pt-Loading) and A_{carbon} are the roughness factor of platinum and carbon in the electrode ($cm_{Pt\text{-}or\text{-}C}^{2}$ cm_{MEA}^{-2}). It is clear that the total capacity charge C_{dl} of UM30 is higher than that of UM50, as both the ECSA of UM30 and the carbon area is higher than those of UM50 (due to the higher carbon loading in UM30). Thus, due to the higher ECSA, Recatalyst PtCo/C should have yielded a higher C_{dl} than UM30 assuming the same carbon area A_{carbon}. However, this is not the case. The lower C_{dl} of ReCatalyst MEA compared to UM30 indicates that A_{carbon} of ReCatalyst PtCo/C has a considerable contribution to the lower C_{dl} of ReCatalyst MEA.

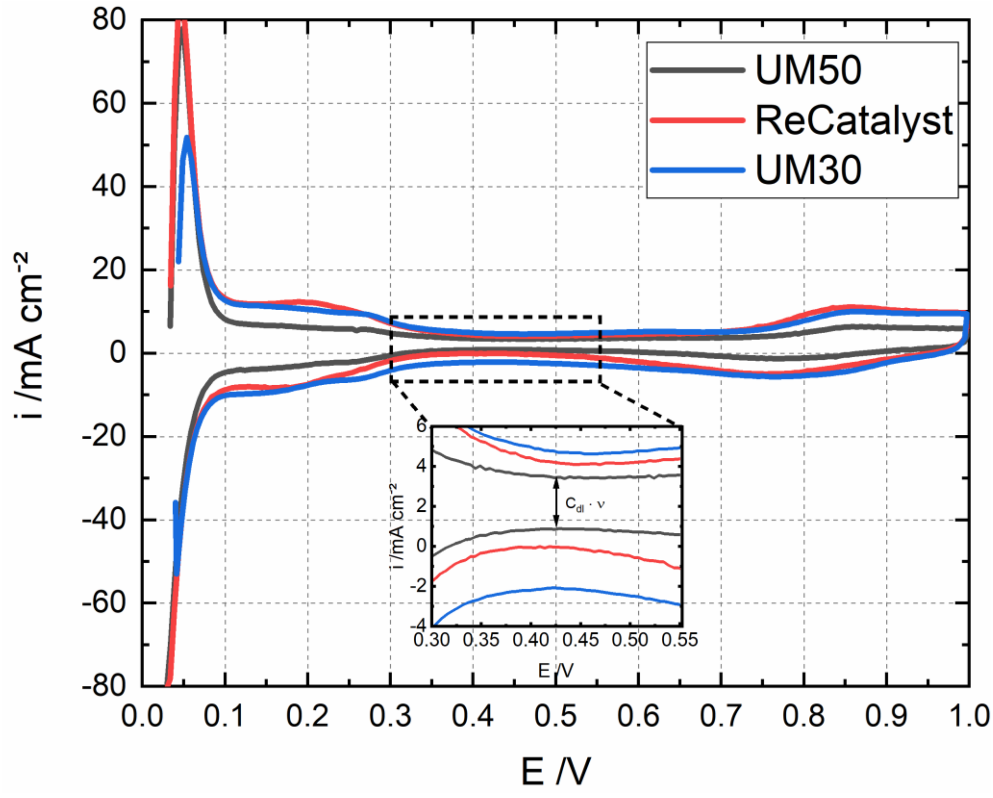

Figure S9: Cyclic voltammograms of MEAs with UM50, ReCatalyst and UM30 Pt-Co/C (0.25 mg_{Pt} cm^{-2}). The double layer region is zoomed-in showing the double layer current density, which is the product of the double layer capacity C_{dl} and the scan rate ν (50 mV s^{-1}).

Tafel analysis

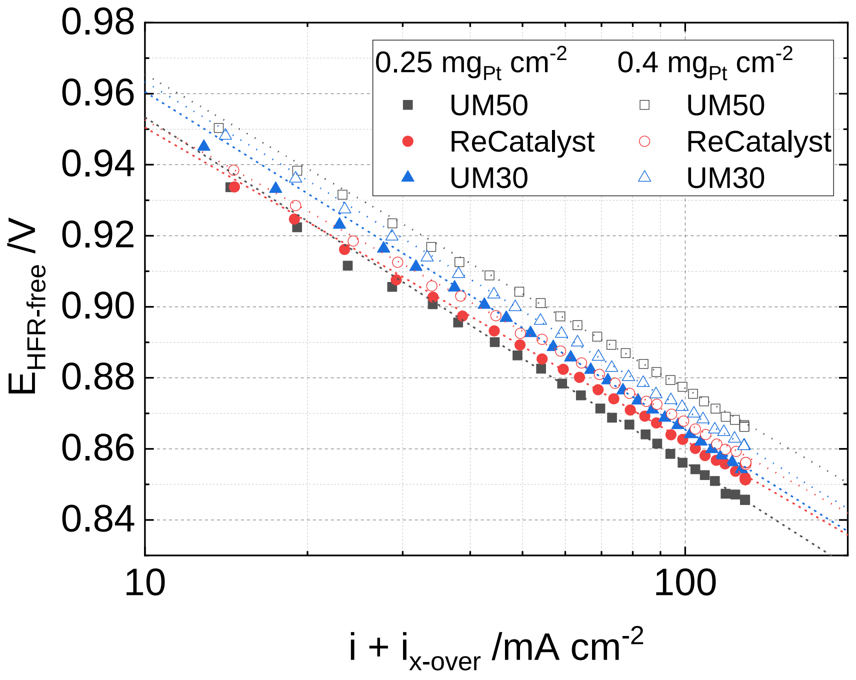

Figure S10: Tafel plots of the three PtCo/C electrocatalysts from two different cathode Pt loadings.

The mass specific current density of UM50 with 0.4 mg_{Pt} cm^{-2} and 0.25 mg_{Pt} cm^{-2} falls on the same line while the deviations of other catalysts (ReCatalyst and UM30) is likely accounted to the greater catalyst layer thickness (> 10 µm, Fig. S12) which could have detrimental effects on the CL utilization.

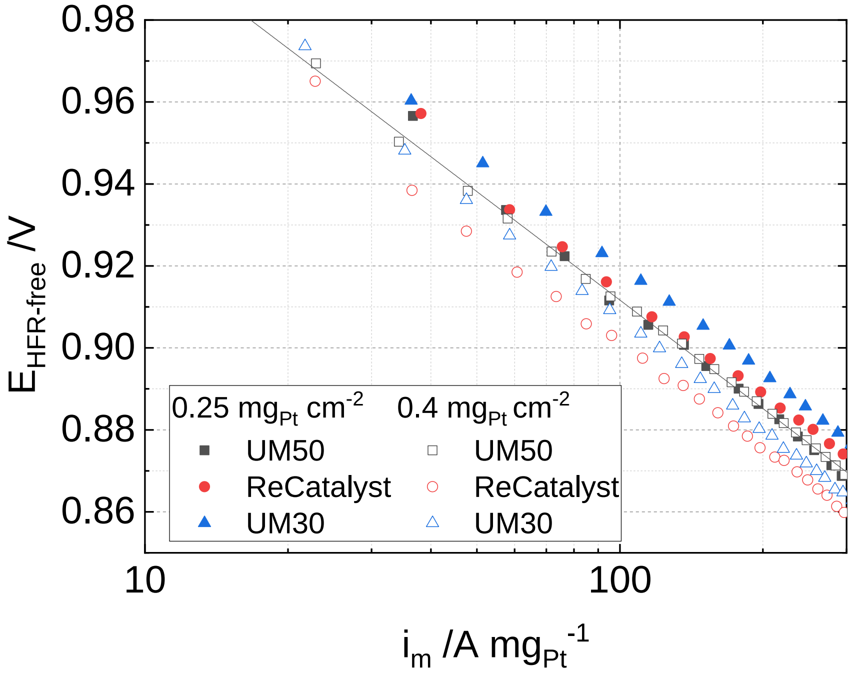

Figure S11: Mass specific current density three PtCo/C electrocatalysts from two different cathode Pt loadings.

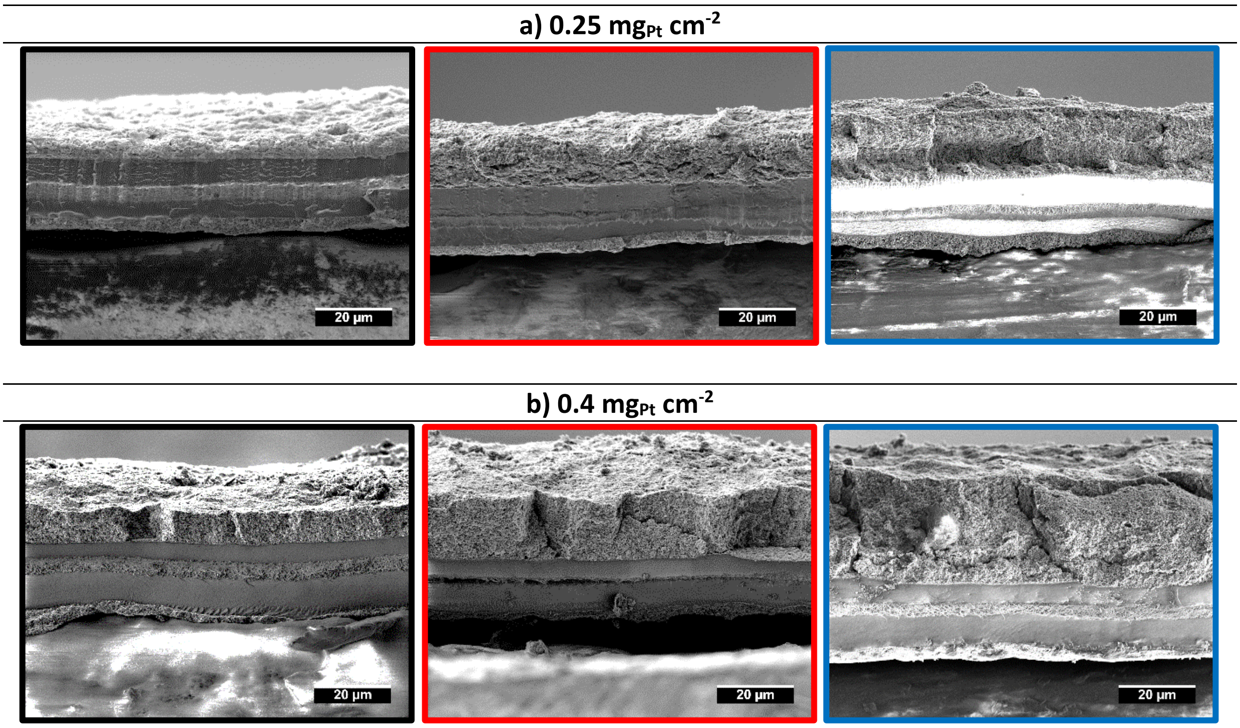

Figure S12: SEM cross sections of the MEAs (FOV = 100 µm) with UM50 (black frame), ReCatalyst (red frame) and UM30 (blue frame) with different cathode Pt loading: a) 0.25 mg_{Pt} cm^{-2} and b) 0.4 mg_{Pt} cm^{-2}. The upmost layer is the cathode catalyst layers, follows by the proton exchange membrane (PEM) with the reinforcement layer. The anode catalyst layer is under the PEM.

It was observed in thin-film rotating disk electrode (TF-RDE) measurements, that the mass activity of ReCatalyst PtCo/C (2 A mg_{Pt}^{-1}) was > 2x higher than the reference catalysts (0.7 A mg_{Pt}^{-1} for UM50 and 0.8 A mg_{Pt}^{-1} for UM30, Table S1).

Table S1: Comparison of electrochemical surface areas (ECSA), mass activities (MA) and specific activities (SA) of the electrocatalysts derived from TF-RDE. The loading was 20 µg for all samples.

**Sample**	**Pt wt%**	**CO-stripping area / cm^2**	**ECSA / m^2 g^{-1}_{Pt}**	**SA@0.90 / mA cm^{-2}_{Pt}**	**SA@0.95 / mA cm^{-2}_{Pt}**	**MA@0.90 / A mg^{-1}_{Pt}**	**MA@0.95 / A mg^{-1}_{Pt}**
**ReCatalyst**	35	6.7	96	2.09	0.27	2.0	0.26
**UM50**	41	4.8	59	~1.2	0.18	0.7	0.10
**UM30**	26	3.5	70	~1.2	0.15	0.8	0.10

Table S2: Mass fraction distribution of the PtCo/C samples derived from TEM measurements.

**Particle diameter / nm**	**Mass fraction for UM50 / %**	**Mass fraction for UM30 / %**	**Mass fraction for ReCatalyst / %**
> 8	25.7	35.5	9.8
7-8	13.1	9.2	6.2
6-7	18.0	13.5	13.0
5-6	22.2	17.6	24.8
4-5	15.2	16.3	28.5
3-4	4.8	7.1	15.5
< 3	0.5	0.8	2.0
